# The puzzle of chloroplast vesicle transport – involvement of GTPases

**DOI:** 10.3389/fpls.2014.00472

**Published:** 2014-09-23

**Authors:** Sazzad Karim, Henrik Aronsson

**Affiliations:** Department of Biological and Environmental Sciences, University of GothenburgGothenburg, Sweden

**Keywords:** cargo protein, chloroplast, clathrin, COPI/II, GTPase, Rab, SAR1, vesicle transport

## Abstract

In the cytosol of plant cells vesicle transport occurs via secretory pathways among the endoplasmic reticulum network, Golgi bodies, secretory granules, endosome, and plasma membrane. Three systems transfer lipids, proteins and other important molecules through aqueous spaces to membrane-enclosed compartments, via vesicles that bud from donor membranes, being coated and uncoated before tethered and fused with acceptor membranes. In addition, molecular, biochemical and ultrastructural evidence indicates presence of a vesicle transport system in chloroplasts. Little is known about the protein components of this system. However, as chloroplasts harbor the photosynthetic apparatus that ultimately supports most organisms on the planet, close attention to their pathways is warranted. This may also reveal novel diversification and/or distinct solutions to the problems posed by the targeted intra-cellular trafficking of important molecules. To date two homologs to well-known yeast cytosolic vesicle transport proteins, CPSAR1 and CPRabA5e (CP, chloroplast localized), have been shown to have roles in chloroplast vesicle transport, both being GTPases. Bioinformatic data indicate that several homologs of cytosolic vesicle transport system components are putatively chloroplast-localized and in addition other proteins have been implicated to participate in chloroplast vesicle transport, including vesicle-inducing protein in plastids 1, thylakoid formation 1, snowy cotyledon 2/cotyledon chloroplast biogenesis factor, curvature thylakoid 1 proteins, and a dynamin like GTPase FZO-like protein. Several putative potential cargo proteins have also been identified, including building blocks of the photosynthetic apparatus. Here we discuss details of the largely unknown putative chloroplast vesicle transport system, focusing on GTPase-related components.

## INTRODUCTION

Eukaryotic cells contain an endomembrane system that delimits organelles with specific functions. These organelles can synthesize various lipids and membrane proteins, but not all of the molecules they require. Thus, diverse lipids, proteins, and hormones are transferred between these organelles, and both to and from the plasma membrane, via small membrane-enclosed sacs called vesicles that bud and dissociate from the donor membrane of one organelle, move then dock and fuse with the acceptor membrane of another organelle (Battey et al., 1999; Lee et al., 2004). Most vesicles have specialized functions related to the cargo they transport (Bonifacino and Glick, 2004; Faini et al., 2013). For instance, some organelles, e.g., lysosomes and vacuoles, host cellular digestion and waste management systems and associated vesicles play key roles in recycling molecules through the endosomal pathway (Dingle, 1968; Mullins and Bonifacino, 2001; Luzio et al., 2007; Grant and Donaldson, 2009). Efforts to elucidate the systems for transporting cargos of vital molecules through the ER, Golgi, secretory granules, and plasma membrane from donor to target membranes were honored with the Nobel Prize in Physiology and Medicine in 2013. More specifically, the prize was awarded to Rothman, Schekman, and Südhof for contributions toward unraveling vesicle transport mechanisms in the yeast *Saccharomyces cerevisiae* and neurotransmitter transport mediated by synaptic vesicles in mammals (Südhof, 2004; Mellman and Emr, 2013).

The possibility that a vesicle-based system may shuttle important molecules between the ER, Golgi, and secretory organelles was initially raised in the 1970s, and vesicle-like structures were first observed in early transelectron microscopic (TEM) studies of pancreatic exocrine cells (Palade, 1975). A general hypothesis was subsequently formulated, postulating that molecules are transported by secretory systems via vesicles formed in a donor membrane then unloaded at a targeted acceptor membrane (Bonifacino and Glick, 2004). Three main classes of vesicles mediating transport have been described since then, based on their protein “coatings:” clathrin-coated vesicle (CCV), coat protein I and II (COPI and COPII) systems (Harrison and Kirchhausen, 2010; Faini et al., 2013). All of these types are morphologically similar, but they have distinct protein and lipid compositions, recognize and transport specific sets of cargo (Rothman, 1994; Schekman and Orci, 1996). CCVs participate in the late secretory pathway, i.e., the endocytic pathway between the Golgi and the plasma membrane (Bonifacino and Glick, 2004); COPI-coated vesicles function in both retrograde (Golgi to ER) and anterograde (within Golgi), while COPII-coated vesicles appear to be involved exclusively in transport from ER to Golgi (Lee et al., 2004; Bethune et al., 2006; Popoff et al., 2011).

Vesicles have also been found in other organelles, including chloroplasts and mitochondria (Morré et al., 1991b; von Wettstein, 2001; Soubannier et al., 2012). Many aspects of the nature and roles of these vesicles remain unknown. However, a decade ago eight putative chloroplast-localized homologs of known protein components of the COPII cytosolic vesicle transport system were identified in the model plant *Arabidopsis* (Andersson and Sandelius, 2004), and the list of COPII-related proteins was recently extended to more than 50 (Khan et al., 2013). Furthermore, putative COPII components were also identified in a recent search for orthologs in other plants, including the agriculturally important *Solanum lycopersicum* (tomato; Paul et al., 2014), and there are experimental indications that two of these proteins are involved in chloroplast vesicle transport (Garcia et al., 2010; Karim et al., 2014). In the following sections we first review current understanding of the three known vesicle transport systems in cytosolic secretory pathways, then apply it to interpret available information on vesicle transport in chloroplasts.

## GENERAL MECHANISMS OF VESICLE CYCLING AND COMPONENTS OF SECRETORY SYSTEMS

Detailed information from yeast and mammalian cells indicate that the general mechanism of vesicle transport involves the following major steps. First, coat assembly is initiated through recruitment of multiple proteins, including membrane-associated small GTPases, transmembrane cargo proteins and Soluble NSF Attachment Protein Receptors (SNAREs) to a donor membrane. Mesh-like vesicles with concentrated cargos then bud through the assembly of coat components, which locally curve the membrane and govern the nascent vesicles’ shape. Following scission from the donor membrane, via the action of accessory proteins, the mature vesicles are uncoated through inactivation of the small GTPases and activation of uncoating enzymes. Coat proteins are recycled for further rounds of vesicle budding, while the naked vesicles proceed to the acceptor membrane, guided by the cytoskeleton, where they are tethered by the combined action of a GTP-bound Rab GTPase and tethering factors. They are then docked through the binding of vesicle (v-) and target (t-) SNAREs (located on the vesicles and acceptor membrane, respectively) and fused with the acceptor lipid bilayer via activation of t-SNARE complexes. Finally, cargo molecules are transferred to the acceptor compartment and the SNAREs are recycled for a new transport round (Bonifacino and Glick, 2004). As already mentioned, three vesicle transport systems in plant cytosol, based on the coat proteins, have been characterized – COPII, COPI, and CCV – all of which are very similar to corresponding systems in yeast and mammals (Kirchhausen, 2000; Bassham et al., 2008). In secretory pathways, vesicles are known to deliver both soluble and membrane-bound proteins to target membranes, leading to the hypothesis that chloroplastic vesicle systems may also deliver proteins in addition to lipids (Khan et al., 2013).

The COPII secretory pathway, which transfers molecules from the ER to the Golgi, has been extensively studied in *S. cerevisiae*. Identified components include five cytoplasmic proteins: Sar1, Sec13, Sec23, Sec24, and Sec31. Each of these proteins has a specific function, and all of them except Sec24 are essential for viability in yeast. In sharp contrast, multiple isoforms of COPII components are present in *Arabidopsis*, and reportedly functional in systematic yeast complementation assays (De Craene et al., 2014). Sar1, a small GTPase of the Arf (ADP-ribosylation factor) GTPase family (Vernoud et al., 2003), is initially recruited to the ER membrane and activated by the ER-associated Sec12, a guanine nucleotide exchange factor (GEF; Barlowe and Schekman, 1993). Sar1 subsequently binds to Sec23, a GTPase activating protein (GAP) that forms a coat subcomplex with a cargo-selecting protein, Sec24 (Bi et al., 2002). Another coat subcomplex consisting of Sec13 and Sec31 binds to the Sec23–Sec24 complex and is thought to be involved in membrane curvature (Lederkremer et al., 2001). Another essential component of the machinery, Sec16, is present at ER exit sites and hypothetically participates in COPII turnover and assembly at transitional ER sites; (Yorimitsu and Sato, 2012; Bharucha et al., 2013).

COPI systems are involved in intra-Golgi and Golgi-to-ER transport. In terms of vesicle formation and transport they are very similar to COPII systems (Kirchhausen, 2000; Trahey and Hay, 2010). The *trans*-Golgi network (TGN)-localized Arf-GEF protein Sec7 can initiate COPI vesicle formation by activating Arf1 (D’Souza-Schorey and Chavrier, 2006), a small GTPase that recruits a heptomeric complex from the cytosol (Orcl et al., 1993; Vernoud et al., 2003). The heptomer consists of two main subcomplexes: the F-COPI subcomplex (with β, γ, ∂, and ζ subunits) and the B-COPI subcomplex (with α, β^-^, and 𝜀 subunits; Fiedler et al., 1996). In contrast to the COPII system, in which the Sar1 GAP is an integral coat component, the Arf1 GTP hydrolysis that induces coat disassembly is mediated by a separate Arf GAP (Poon et al., 1999).

CCV systems, which are more complex than COPII and COPI systems, mediate plasma membrane endocytosis and transport from the TGN to endosomes and lysosomes (Trahey and Hay, 2010). In CCV clathrin heavy chain (CHC) and clathrin light chain (CLC) coat proteins form a geometrical scaffold, a triskelion, around the vesicle membrane (Kirchhausen and Harrison, 1981), and they are associated to adaptor protein (AP) complexes to bind to the membrane components. Similar to COPI, Arf GTPases are involved in the recruitment of a variety of coat subunits adaptor protein (AP) complexes. Five types of AP complexes (AP1 to AP5), involved in different pathways, have been recognized (Hirst et al., 2011). AP1 found on the TGN and endosomes, and AP2 found on the plasma membrane (Keen, 1990), attach clathrin to the donor membrane, select the protein cargo and recruit accessory proteins that regulate vesicle formation. AP3 and AP4, are also present on the TGN and endosomal membranes, but AP3 is mainly localized to endosomes and AP4 mainly to the TGN (Robinson and Bonifacino, 2001). AP4, and the fifth complex (AP5, localized to late endosomes) apparently participate in the late endosomal pathway (Hirst et al., 2011, 2013; Barlow et al., 2014). The *Arabidopsis* genome contains genes encoding putative members of all five classes (Bassham et al., 2008; Yamaoka et al., 2013). In both COPI- and CCV-mediated transport ARF GTPases are involved in the budding of vesicles from donor membranes (Memon, 2004).

Membrane fusion at the target membrane is mediated by the interaction of soluble SNARE proteins, with other proteins involved in the process, through their highly conserved SNARE domains. SNAREs are classified into two types: v-SNAREs (acting on the vesicle) and t-SNAREs (acting on the target membrane). During the membrane fusion one v-SNARE and three t-SNAREs interact to form a trans-SNARE complex in the vicinity of the target membrane. According to the amino acid residues glutamine (Q) or arginine (R) in the so-called 0-layer of the SNARE motif, SNAREs are divided into Q- and R-SNARES. The Q-SNAREs is further divided into three subgroups (Qa, Qb, and Qc) depending on their locations in the core complexes, and in the R-SNARE glutamines are coordinated through hydrogen bonding to an arginine residue (Palfreyman, 2009; Kim and Brandizzi, 2012; El Kasmi et al., 2013).

Tethering factors form a bridge between vesicles and the target membrane and interact with Rab GTPases and SNAREs to ensure fusion. Among the two main classes of tethering factors one class is the oligomeric complexes such as conserved oligomeric Golgi (COG) complex and Exocysts that bind to SNARES and act as RAB effectors or oligomeric complexes such as transport protein particle I and II (TRAPP I and TRAPP II) complexes that act as GEFs for Rabs. The other class of coiled-coil tethering factors that function as Rab effectors or Rab GEFs (Eckardt, 2008; Sztul and Lupashin, 2009; Thellmann et al., 2010; Khan et al., 2013). A bioinformatics study predicted several tethering factors to exist in *Arabidopsis* chloroplast, e.g., COG complexes, Exocysts, and AtCASP that could be dual localized having role in CPOII mediated transport but no homologs for TRAPP complexes were found in the chloroplast (Khan et al., 2013).

## ORIGINS OF THE CHLOROPLAST VESICLE TRANSPORT SYSTEM

A vesicle transport system may also be present in cyanobacteria, since photosynthetic membrane-bound vesicles (Nevo et al., 2007) and vesicle-like structures between their well separated plasma membrane and thylakoids (Schneider et al., 2007) have been observed in TEM images. Furthermore, Tvp38 a SNARE-associated protein with predicted chloroplast localization (Khan et al., 2013), is conserved in chloroplasts and cyanobacteria (Keller and Schneider, 2013), and several of the proteins with suggested involvement in chloroplast vesicle transport are also present in cyanobacteria. Thus, as plant chloroplasts are believed to have arisen from bacterial endosymbiosis related to cyanobacteria engulfed by a eukaryotic cell (Palmer, 2000). Interestingly, VIPP1, responsible for vesicle formation, has been identified in both cyanobacteria and *Arabidopsis*, which suggests that vesicle transport in chloroplasts originated from a prokaryotic origin such as cyanobacteria, and thus vesicle transport systems could have existed in cyanobacteria before embryophytes emerged (Westphal et al., 2001a).

However, similarities with the cytosolic system suggest that the chloroplast transport machinery has eukaryotic origins, possibly via transfer of components of the secretory pathway to the chloroplast followed by divergent evolution (Vothknecht and Soll, 2005). Analysis of organisms from diverse lineages has revealed that vesicle transport in chloroplasts only occurs in embryophytes, indicating late evolutionary acquisition by land plants (Westphal et al., 2003). This may reflect intense selective pressure to evolve sophisticated systems for constructing, maintaining, and modulating more complex thylakoids following the transition from an aqueous to a non-aqueous environment.

It is possible that some proteins involved in chloroplast vesicle transport have eukaryotic origins while others are prokaryotic. Their roles in chloroplast vesicle transport have not been clearly established, but the latter could have been integrated with a vesicle system transferred from the cytosol to replace existing eukaryotic equivalents. Proteins with no functional homologs in evolving chloroplasts may have been imported from the cytosol, while prokaryotic proteins capable of substituting for cytosolic proteins may have been functionally modified.

### SUPPORT FOR A VESICLE TRANSPORT SYSTEMS IN CHLOROPLASTS

Plant chloroplasts are surrounded by two lipid-bilayer membranes (the outer and inner envelopes) separated by an inter-membrane space. The stroma, the main aqueous compartment of the organelle, contains stacks of thylakoids, sites of photosynthesis. Thylakoids are lipid bilayers with a unique glycerolipid composition and significant proportions of both embedded and peripheral multi-protein complexes. They are composed primarily of phospholipids and galactolipids; mono-, and digalactosyldiacylglycerol represent ∼50 and ∼25 mol% of the total thylakoid lipids, respectively (Douce and Joyard, 1990). Thylakoids are unable to synthesize these compounds. Instead they are produced through a complex pathway involving exchange of lipid precursors between the ER and the inner envelope. Therefore, as the final production site (the inner envelope membrane) and destination (thylakoid) membrane are separated by an aqueous stroma, a specific transport system for these highly hydrophobic compounds is presumably required. Several theories have emerged to explain the transport of hydrophobic compounds to the thylakoids, including lipid transfer at sites of physical contact, diffusion of monomers facilitated by lipid transfer proteins and a vesicular mechanism. However, while there is no evidence for sites of physical contact or lipid transfer proteins, ultrastructural and biochemical studies corroborate the “vesicle theory” (Andersson and Sandelius, 2004).

Vesicle-like structures in plastids were observed in early TEM studies under certain conditions (Muehlethaler and Frey-Wyssling, 1959; von Wettstein, 1959). These structures appear in the stroma between the chloroplast envelope and thylakoid after low temperature incubation of leaf tissues (Morré et al., 1991b; Westphal et al., 2001b, 2003). Thin serial TEM sections demonstrate that the accumulated vesicles are not tubular extensions from the inner envelope but membrane-enclosed sacs with estimated diameters of 30–70 nm (Westphal et al., 2001b). Vesicles attached to the inner envelope membrane and the thylakoid can also be observed, suggesting the occurrence of fission and fusion events. The appearance of vesicles solely at low temperature (4°C) suggests that they could be artifacts generated, for instance, when preparing samples for TEM examination. However, analyses of isolated chloroplasts incubated with radiolabeled lipid precursors have shown that galactolipid synthesis *in vitro* is also temperature-dependent. Furthermore, radiolabeling is detectable in lipids in the inner envelope before it appears in thylakoid membranes, strongly indicating that lipids synthesized in the chloroplast envelope are transported to the thylakoid (Andersson et al., 2001). These observations suggest that synthesis and transport of galactolipids, as well as the associated vesicle fission and fusion, can only be observed with current techniques at low temperature because they are too rapid at normal growth temperatures. The cytosolic vesicle transport system between the ER and the Golgi apparatus in mammalian cells can also be blocked by exposure to low temperature, suggesting a certain analogy between the two systems (Moreau et al., 1992).

In support of this analogy, inhibitors of vesicle transport via the secretory pathway have similar inhibitory effects on chloroplast vesicle formation, as demonstrated by the following observations (Westphal et al., 2001b). Addition of *o*-GTP, GTPγS, and GMP/PNP, which are non-hydrolysable nucleotide analogs, results in nearly complete loss of vesicle accumulation in isolated chloroplasts incubated at 4°C, and AlF^-^_4_ (an inhibitor of GTP-binding proteins) is even more strongly inhibitory. This indicates that vesicle budding is controlled via a GTPase, as in the cytosolic vesicle system where several GTPases are involved in distinct steps of vesicle transport.

The addition of microcystin LR, which impairs membrane fusion in vacuole formation by inhibiting protein phosphatase 1, induces accumulation of vesicles in isolated chloroplasts, suggesting that a protein phosphatase might also be involved in their fusion. Ophiobolin A, an inhibitor of calmodulin, and W7, a Ca^2+^ antagonist, can also inhibit fusion of vesicles with the acceptor membrane (Westphal et al., 2001b). This is a further similarity, as Ca^2+^ and calmodulin participate in membrane fusion in the secretory pathway (Peters and Mayer, 1998; Mayer, 1999). Furthermore, investigations using a cell-free system prepared from isolated chloroplasts have revealed requirements for stromal proteins and ATP for transport of lipids to thylakoids, and for stromal proteins, ATP and GTP for their release from isolated envelopes. Their release is also stimulated by acyl-CoA (Morré et al., 1991a; Räntfors et al., 2000).

Collectively, these observations strongly suggest the presence of a protein-mediated vesicle transport system in chloroplasts with basic components similar to those of cytosolic vesicle transport systems.

## EXPERIMENTALLY IDENTIFIED MOLECULAR COMPONENTS OF CHLOROPLAST VESICLE TRANSPORT

**FIGURE 1:**
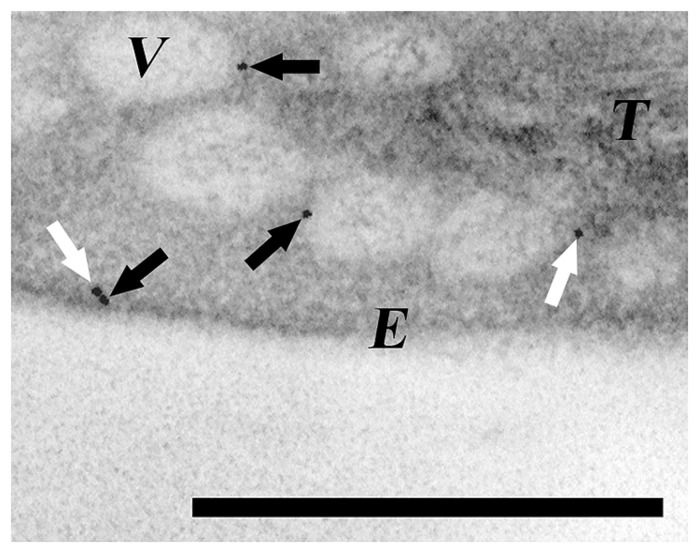
**Detection of CPSAR1 in *Arabidopsis* chloroplasts.** Transmission electron micrograph of immunogold-labeled sections of leaves after low temperature incubation (Garcia et al., 2010) showing the presence of CPSAR1 in the stroma (white arrow) and at both the envelope and vesicle membranes (black arrows). E, envelope; V, vesicle; T, thylakoids. Bar 0.5 μm.

If the putative chloroplast vesicle transport system is related to cytosolic systems there is probably some conservation of components between them (Andersson and Sandelius, 2004). Accordingly, using the *Arabidopsis* genome and web-based localization prediction tools chloroplast-localized proteins with high sequence similarity to components of cytosolic systems have been identified, including eight putative COPII-related vesicle transport proteins (Andersson and Sandelius, 2004). A recent bioinformatics study confirmed the previously reported COPII-related proteins, mainly involved in the initiation and budding stages, and identified several further putative components, e.g., SNAREs, Rabs, tethering factors, and reticulons that could collectively form a complete vesicle transport system in chloroplasts (Khan et al., 2013). Interestingly, two putative components identified in bioinformatics analyses (Andersson and Sandelius, 2004; Khan et al., 2013) have been confirmed to be chloroplast localized, and like the homologous cytosolic vesicle components shown to be active GTPases: CPSAR1 (**Figure 1**) and CPRabA5e (CP, chloroplast localized) (**Table 1**; Garcia et al., 2010; Karim et al., 2014). The following sections discuss possible roles of these and several other potentially important players in chloroplast vesicle transport.

**Table 1:** Chloroplast localized proteins putatively involved in chloroplast vesicle transport.

Protein, AGI No.	Chloroplast localization	Mutant phenotype	Chloroplast phenotype	Domain structures	Reference
CPSAR1, At5g18570	IEM^1^, stroma, vesicles	Embryo-lethal, non-viable	Absence of thylakoid membranes, presence of stromal vesicles	TP^2^, coiled-coil domain, GTPase domain	Bang et al. (2009), Chigri et al. (2009), Garcia et al. (2010)
CPRabA5e, At1g05810	Stroma, thylakoid	No visible phenotype	Altered thylakoid development and organization, increased presence of stromal vesicles	TP^2^, GTPase domain, geranylgeranylation motif, unique YYRGA motif	Karim et al. (2014)
THF1, At2g20890	Envelope, stroma, thylakoid	Variegated leaves, delayed development	Normal green sectors in the chloroplast, but absence of thylakoid membranes and presence of stromal vesicles in white sectors	TP^2^, coiled-coil domain	Wang et al. (2004), Huang et al. (2006)
VIPP1, At1g65260	IEM^1^, thylakoid	Pale-green leaves, deficient photosynthesis	Balloon like structure, absence of thylakoid membranes, presence of stromal vesicles	TP^2^, α-helical domain, plant specific C-terminal domain	Kroll et al. (2001), Aseeva et al. (2007), Zhang et al. (2012), Otters et al. (2013)
FZL, At1g03160	IEM^1^, thylakoid	Pale-green leaves, delayed development	Fewer and larger chloroplasts, disorganized thylakoid array, fewer stroma thylakoids	TP^2^, coiled-coil domain, GTPase domain, TM^3^ domain	Gao et al. (2006)
SCO2/CYO1, At3g19220	Thylakoid	Pale green/albino cotyledon	Vesicles emerged from IEM^1^ mainly at rounded ends of elongated chloroplasts of embryonic leaves	Zn-finger domain	Shimada et al. (2007), Muranaka et al. (2012), Tanz et al. (2012)
CURT1 (A-D), At4g01150, At2g46820, At1g52220, At4g38100	Thylakoid	No visible phenotype	Flat lobe-like thylakoids with fewer grana margins	TP^2^, two TM^3^ domains	Armbruster et al. (2013)

*^1^Inner envelope membrane.*

*^2^Transit peptide.*

*^3^Transmembrane.*

### GTPases

#### Small GTPase CPRabA5e

As discussed above, small GTPase Rab proteins regulate almost all cytosolic membrane trafficking steps, from the budding of transport vesicles at the donor membrane to their fusion with the target membrane. In their regulatory cycle Rabs select cargo, promote vesicle movement to specific membranes and verify the correct site of fusion via interactions with diverse effector proteins. Rabs generally have a characteristic GTPase fold, composed of a six-stranded β-sheet flanked by five α-helices. The presence of multiple Rabs in the organisms proteome suggests they play specific transport roles in specific pathways (Hutagalung and Novick, 2011; Li et al., 2014). GTPase Rabs have both GTP/GDP binding and GTP hydrolysis capability, allowing them to act as molecular switches: active when GTP-bound and inactive when GDP-bound (Takai et al., 2001; Agarwal et al., 2009). A Rab escort protein (REP) binds newly synthesized Rab proteins and presents them to geranylgeranyl transferase for geranylgeranylation/prenylation at their two C-terminal cysteines, which increases their hydrophobicity, before targeting them to the membrane delivery system (Leung et al., 2006; Schwartz et al., 2007). Prenylated Rabs are stabilized in their inactive, GDP-bearing conformation by binding to the GDP dissociation inhibitor (GDI; Stenmark, 2009; Pfeffer, 2012). The GDI displacement factor (GDF) catalyzes dissociation of Rab–GDI complexes, thereby facilitating association of the Rabs with appropriate membranes. At their respective target membranes Rabs are activated by binding GTP with the assistance of a GEF. Upon completion of a transport cycle GTP hydrolysis is triggered by GAPs, returning the Rabs to an inactive GDP-bound state, which again is maintained by GDI until a new transport cycle is initiated by their recruitment to a membrane (Ali and Seabra, 2005; Nielsen et al., 2008; Stenmark, 2009).

**FIGURE 2:**
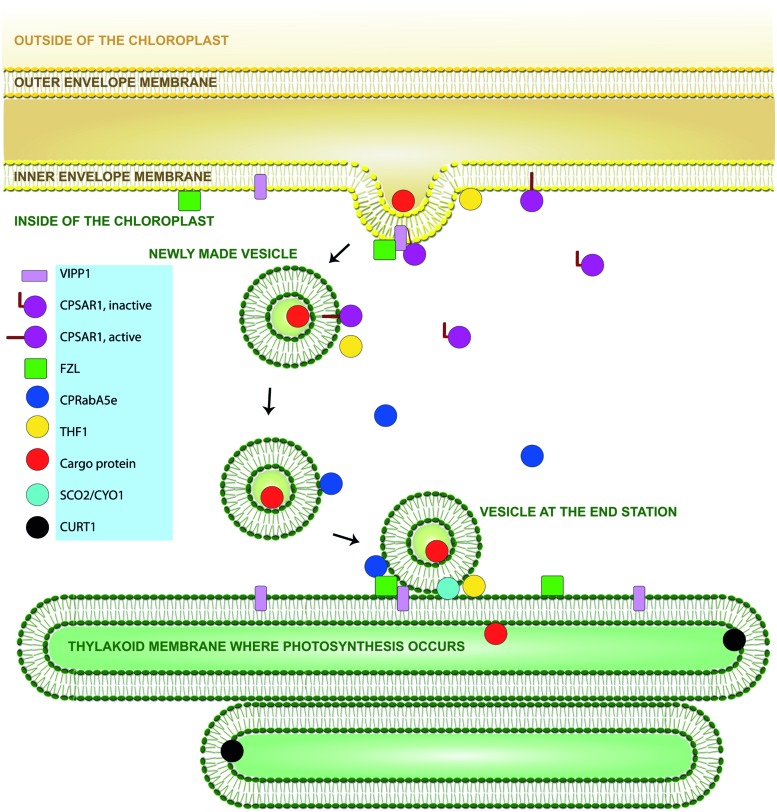
**Schematic diagram of vesicle formation and movement in chloroplasts.** Proteins included are those with suggested involvement in chloroplast vesicle transport, and verified to be chloroplast localized. VIPP1 (pink), FZL (green), and THF1 (yellow) are located both at the donor membrane (inner envelope) and the acceptor membrane (thylakoids), and could thus participate in both fission and fusion of vesicles. CPSAR1 (violet) is located at the donor membrane, the vesicle and in the stroma, indicating that it plays a similar role as the cytosolic Sar1, i.e., in an active GTP-bound state it helps to form vesicles, and in an inactive GDP-bound state it is recycled back to the donor membrane for a subsequent cycle. THF1 (yellow) and CPRabA5e (blue) are both located in the stroma and the acceptor membrane (thylakoids) may interact with the vesicle just prior to fusion and facilitating this step. SCO2/CYO1 (turquoise) and CURT1 (black) are located at the acceptor membrane and may interact with vesicles for the transport of molecules, e.g., cargo protein(s; red) and facilitating the fusion (delivery) step.

CPRabA5e (**Figure 2** and **Table 1**), one of the recently identified chloroplast-localized Rab GTPases in *Arabidopsis* (detected by immunoblotting in both the stroma and thylakoids; Karim et al., 2014), is a close homolog of Ypt31/32 GTPases in yeasts. These proteins are involved in trans Golgi trafficking and required for recycling proteins (such as work together with effector Rcy1 for recycling of vSNARE Snc1 in yeast) from the plasma membrane through early endosomes to the Golgi complexes (Chen et al., 2011); putatively participate in the yeast exocytic pathway; and apparently involved in both Golgi-to-plasma membrane and endosome-to-Golgi transport (Jedd et al., 1997; Zou et al., 2012). The Ypt31p/32p pathway may also regulate putative phospholipid translocases that promote formation of vesicles destined for the TGN, and are thought to be involved in the generation of phospholipid asymmetry in membrane bilayers (Furuta et al., 2007). CPRabA5e restores growth of *ypt31Δ ypt32^ts^* mutants at 37°C in yeast complementation studies, strongly supporting the bioinformatic indications that CPRabA5e participates in vesicle transport. In addition, seed germination is delayed and under oxidative stress growth is arrested in *cprabA5e* knockout mutants, chloroplasts contain larger plastoglobules, thinner grana, and more vesicles close to the envelopes than wild type counterparts (**Table 1**). A yeast-two-hybrid screen with CPRabA5e as bait revealed 13 interacting proteins, mainly located in thylakoids and plastoglobules. These proteins have known or predicted involvement in development, stress responses, and photosynthesis related processes, consistent with the observed stress phenotypes. They include curvature thylakoid 1A (CURT1A), one of a set of four CURT1 and proteins (A–D) that are conserved in plants and cyanobacteria, highly concentrated at grana margins of thylakoids (**Figure 2** and **Table 1**). In a recent work it has been shown elaborately that the CURT1 proteins (A–D) as a family cluster form oligomers, control the membrane curvature, determine the architecture of grana and control grana formation in *Arabidopsis* (Armbruster et al., 2013). Interestingly, in double, triple, and quadruple loss of function mutants of CURT1 (A–D) thylakoids were occasionally wider and curved, lacking grana stacks and accumulating vesicles, suggesting a role of theses vesicles in thylakoid formation (Armbruster et al., 2013; Luque and Ochoa de Alda, 2014). However, both *curt1a* single mutant and *cprabA5e* mutant chloroplasts reportedly have thin grana, supporting the notion that CPRabA5e is involved in processes that control thylakoid morphology (Armbruster et al., 2013; Karim et al., 2014).

In addition, two other putatively chloroplast-localized Rab proteins have been identified, RabB1c and RabF1. The latter (also known as ARA6 in *Arabidopsis*, or RAB5 according to human Rab nomenclature) is unique to plants, and reportedly involved in endosomal transport and modulation of the assembly of SNARE-related complexes (Ebine et al., 2011, 2012). Preliminary data obtained using an ARA6/RabF1 specific antibody have verified the presence of CPRabF1 in both the envelope and thylakoids of chloroplasts, implying that it has dual localization (Alezzawi, 2014). However, the precise role of CPRabF1 in vesicle transport in chloroplasts requires further elucidation. CPRabF1’s localization in the envelope and thylakoid membranes could indicate that CPRabF1 (ARA6) in the vicinity of the donor (envelope/thylakoid) and acceptor (envelope/thylakoid) membranes might modulate assembly of SNARE complexes in chloroplasts (Ebine et al., 2011, 2012). However, further verifications of this hypothesis are required.

#### GTPase CPSAR1

CPSAR1 (**Figures 1** and **2**; **Table 1**) is suggested to be a homolog of Sar1, which is essential for assembly of the COPII coat. It has GTPase activity, plays a role in thylakoid biogenesis, and is present both at the inner envelope and in the stroma (compatible with a function in vesicle initiation), but also around cold-induced vesicles (**Figure 1**; Garcia et al., 2010). Several GTPases, including the cytosolic Arf1 and Sar1, have dual distributions being located at donor membranes when GTP-bound and soluble following GTP hydrolysis (**Figure 2**). Moreover, *cpsar1* knockout in *Arabidopsis* causes severe disorders, including embryo lethality and absence of thylakoids in embryos, signifying a role in thylakoid biogenesis (**Table 1**). CPSAR1 is a member of the Obg (Spo0B-associated GTP-binding protein) subfamily, which has suggested involvement in various processes, including chromosome partitioning, ribosome functions, sporulation, and stress responses (Kobayashi et al., 2001). Accordingly, CPSAR1 has proposed roles in ribosome biogenesis involving chloroplast-encoded subunits, and thus has also been named ObgC (Bang et al., 2009, 2012). Thus, there is evidence for two distinct roles of CPSAR1. However, the presence of CPSAR1 in envelopes and vesicles, but not close to the thylakoids where ribosomes are closely located, indicates that it is functionally involved in vesicle transport (Marin-Navarro et al., 2007).

#### Large GTPase dynamins

Dynamins comprise a large superfamily of GTPases with five characteristic domains. Dynamins are involved in many cellular processes, *inter alia* division of chloroplasts, peroxisomes and mitochondria. They also participate, in concert with clathrin assembly and membrane-remodeling proteins, in the invagination of clathrin-coated buds and vesiculation during clathrin-mediated endocytosis (CME), which plays various critical roles in plant development. Dynamin-related proteins (DRP) differ from classical dynamins, but share at least three of the five characteristic domains; the GTPase, middle, and GTPase-effector domain (GED; Heymann and Hinshaw, 2009). In plants there are two main families of DRPs, both of which apparently participate in clathrin-mediated transport, DRP1 and DRP2. The *Arabidopsis* genome encodes 16 DRPs, grouped into six subfamilies (DRP1–DRP6) based on their amino acid sequences and predicted domains (Backues et al., 2010; Bednarek and Backues, 2010). DRP 5B, DRP5B/ARC5 (accumulation and replication of chloroplasts 5) is a functional GTPase, essential for the division of chloroplasts in plants and localized at the outer envelope of chloroplasts. DRP5B/ARC5 has two functional isoforms and plastid division proteins 1 and 2 (PDV1 and PDV2) interact with DRP5B/ARC5 and regulate its GTPase activity (Gao et al., 2013; Holtsmark et al., 2013).

Several functional analogs of dynamins such as a filamentous temperature sensitive z protein, FtsZs (tubulin-like GTPases) play essential roles in the division of prokaryotic and eukaryotic cells, chloroplasts, and mitochondria. In plants, stromal FtsZ ring (Z-ring) formation initiates the division process. *Arabidopsis* harbors three FtsZ genes: FtsZ1, FtsZ2-1 (which are functionally redundant; Schmitz et al., 2009) and FtsZ2-2. The Z-rings of FtsZs can spontaneously self-assemble and interact with each other (El-Kafafi et al., 2008; Gargano et al., 2013). FZO is a dynamin-related membrane-remodeling protein that mediates fusion between mitochondrial outer membranes in animals and fungi. A single FZO-like protein in *Arabidopsis*, FZL, is regarded as a plant-specific member of the dynamin superfamily associated with the thylakoid and envelope membranes (**Figure 2** and **Table 1**). *Fzl* mutants have abnormalities in both morphology and the distribution of granal and stromal thylakoids (**Table 1**). In *fzl* mutant chloroplasts grana lamellae are less uniform in shape and inflated at their margins, resulting in a disorganized thylakoid structure and accumulation of vesicles, indicating that theses vesicles have a role in thylakoid morphology (Gao et al., 2006). FtsZ1 and FZL, which are involved in plastid division, participate in remodeling of thylakoid membranes and are not involved in early steps of thylakoid biogenesis. FtsZ phosphorylation affects GTPase activity, polymerization, and interactions with other division proteins. Interestingly, AtFtsZ2 is phosphorylated *in vivo* and it has been suggested that an interacting partner of AtFtsZ2, phosphoglycerate kinase 1 (PGK1), might be responsible for FtsZ phosphorylation. This phosphorylation may play a regulatory role in plastid division (Gargano et al., 2012). PGK1 is reportedly an interacting partner of CPRabA5e, but it is unclear whether CPRabA5e participates in PGK1 regulation. If so, chloroplast-localized Rabs could be linked to plastid division through remodeling of the thylakoid membranes. However, in *cprabA5e* mutants the granal stacks are reportedly appressed to thylakoid membranes (Karim et al., 2014).

Dynamin-related proteins ADL1A/DRP1A and ADL2A/DRP3A (role in both peroxisomal and mitochondrial division; Zhang and Hu, 2010) in *Arabidopsis* appear to be similar to the animal dynamin I protein, both subfamilies seems to be unique to plants (Park et al., 1997; Kang et al., 2003; Fujimoto et al., 2010). Analysis of *Arabidopsis adl1A* mutants showed ADL1A to be vital role for embryogenesis, seedling development, reproduction, formation of Golgi stacks and accumulation of secretory vesicles close to the plasma membrane (Park et al., 1997). In absence of ADL1A abnormal accumulation of plasma membrane possibly disrupts targeting and fusion of exocytotic vesicles to the cell surface as a result disruption of cell wall biosynthesis occurs (Kang et al., 2003). *Arabidopsis* ADL1A deletion mutants showed yellow leaf phenotype with reduced number of chloroplast, not morphologically developed with reduced amount of thylakoid membranes. In earlier reports the localization of ADL1A in thylakoid membranes in suborganellar fractionation suggested ADL1A to have a role in vesicle formation as a dynamin protein in chloroplasts (Park et al., 1998). However, in contradiction a later study including subcellular fractionation and immunolocalization results obtained by using the ADL1A-specific antibody established that ADL1A is targeted to the cell plate during cytokinesis and not being associated with chloroplasts (Kang et al., 2001). Moreover, using GFP fusion proteins it was also observed that the ADL2A was localized in mitochondria but not in chloroplasts (Arimura et al., 2004). Therefore it raises doubts whether or not ADL1A and/or ADL2A are CP localized.

### OTHER IMPORTANT FACTORS FOR CHLOROPLAST VESICLE TRANSPORT

#### Vesicle-inducing protein in plastids 1 (VIPP1)

The *Arabidopsis* vesicle-inducing protein in plastids 1 (VIPP1) is localized in the inner envelope and the thylakoid membranes even though VIPP1 is a hydrophilic protein (**Figure 2** and **Table 1**). In chloroplasts of *vipp1* deletion mutant vesicle formation is abolished, thus it results in inhibition of thylakoid formation (Kroll et al., 2001). VIPP1 is closely related to the phage shock protein A (PspA) in *Escherichia coli*, a protein induced under diverse stress conditions. In addition, VIPP1 possesses an additional plant specific C-terminal domain, suggested to be required for its role in thylakoid biogenesis (Aseeva et al., 2004). VIPP1 retains α-helical domain same as in PspA, which is essential for the association of VIPP1 to the inner envelope membrane (Otters et al., 2013). However, VIPP1 is essential for both thylakoid formation and for the maintenance of chloroplast envelopes. The knockout mutant of VIPP1 is lethal but knock-down or partial mutant plants with a reduced amount of VIPP1 showed variegated leaves (pale-green) and cotyledons, whereas young seedlings of knockout mutants can only survive on MS medium showing albino morphology. Knock-down plants showed decrease in thylakoid membrane content, but thylakoid membranes are assembled with thylakoid protein complexes being photosynthetically active. It suggests that VIPP1 is required for basic thylakoid membrane formation but not being involved in the content of thylakoid protein complexes (Westphal et al., 2001a; Aseeva et al., 2007; Vothknecht et al., 2012; Zhang et al., 2012). VIPP1 has also been suggested to be involved in protein translocation by enhancing protein transport through the twin-arginine translocation (Tat) pathway (Lo and Theg, 2012). Moreover, the *vipp1* knockdown and knockout plants show balloon-like structures in chloroplasts, a rather unique morphology. All *vipp1* knockout mutant plastids demonstrated balloon-like chloroplasts without any green area (thylakoid membranes) whereas in knockdown plants many of the chloroplasts show balloon-like structures containing thylakoids in a limited area of the chloroplasts (Zhang et al., 2012).

#### Thylakoid formation 1 (THF1)

An *Arabidopsis* thylakoid formation1 (THF1) protein is found to be localized in the envelope, thylakoid membrane and stroma of chloroplasts (**Figure 2** and **Table 1**; Wang et al., 2004; Huang et al., 2006). THF1 was suggested to be involved in the process of organization of transport vesicles into mature thylakoid stacks. The *thf1* mutant showed variegation (pale green cotyledons) phenotype in both cotyledons and leaves, slower growth rates, defects in etioplast development in darkness, increased sensitivity to high light, lower PSII efficiency, and stay-green phenotype in stress-induced leaves (Wang et al., 2004; Keren et al., 2005; Wu et al., 2011; Huang et al., 2013; Gan et al., 2014). Plastids of pale/non green sectors of variegated leaves of *thf1*, which are suggested to be light independent, accumulate membrane vesicles and lack organized thylakoid structures, suggesting that THF1 plays a vital role in a process of vesicle-mediated thylakoid membrane biogenesis (Wang et al., 2004; Wu et al., 2011). THF1 was found to be controlling photosystem II–light-harvesting complexes (PSII–LHCII) dynamics during dark induced senescence and light acclimation. It interacts with the light-harvesting chlorophyll a/b binding protein LHCB in a pH-dependent manner, and the stay-green phenotype of *thf1* relies on the presence of LHCII complexes (Huang et al., 2013). In etioplasts of dark-grown cotyledons of *thf1* mutants ultrastructural analysis identified coexistence of plastids with different developmental stages of prolamellar bodies (PLB), i.e., from normal developed to almost no PLB developed, and chlorophyll biosynthesis and expression of several plastidic genes were suppressed in *thf1* (Wu et al., 2011). Interestingly, the THF1 ortholog in rice is suggested to be involved in the degradation of chlorophyll (Yamatani et al., 2013). In addition, THF1 protein has also been proposed to have a role in sugar signaling in *Arabidopsis* through the interaction with a heteromeric G-protein GPA1 (Huang et al., 2006).

#### Snowy cotyledon 2/cotyledon chloroplast biogenesis factor (SCO2/CYO1)

An *Arabidopsis* disulfide isomerase located in thylakoids (**Figure 2** and **Table 1**), SCO2/CYO1, caused specifically disruption of chloroplast biogenesis in cotyledons in *sco2/cyo1* mutants. The *sco2*/*cyo1* mutants showed pale green/albino cotyledon but turned into normal green true leaves during development (Shimada et al., 2007; Albrecht et al., 2008; Muranaka et al., 2012; Tanz et al., 2012). chloroplasts ultrastructure observed in *sco2/cyo1* cotyledons were either of normal shape or were globular with large vesicles emerged from the inner envelope mainly at the rounded ends of the elongated chloroplasts, not observed in wild-type chloroplasts. Loss of SCO2/CYO1 might interrupt the functions of vesicles formation for thylakoid membranes in embryonic leaves and as a result accumulation of these vesicles was apparent at the rounded ends of the *sco2/cyo1* chloroplasts (Tanz et al., 2012). These vesicles were involved in the integration of the photosystem proteins into thylakoids including LHCB. SCO2/CYO1 was found to be interacting with the protein subunits of PSI and PSII (Muranaka et al., 2012; Tanz et al., 2012). It was observed that LHCB1 interacted with SCO2/CYO1 thus, SCO2/CYO1 might be involved in integration of LHCB1 proteins into thylakoids. Interestingly, this suggests that the lack of interaction of SCO2/CYO1 with LHCB1 in *sco2/cyo1* mutants results in inhibiting integration of LHCB, delaying the merging of vesicles into thylakoid membranes and thus vesicles being accumulated in chloroplasts (Tanz et al., 2012).

## VESICLE TRANSPORT OF CARGO PROTEINS IN CHLOROPLASTS

An intriguing question is whether proteins are also transported in vesicles to the thylakoids in addition to lipids (**Figure 2**). No cargo proteins have been definitively identified, but downregulation of several nuclear-encoded photosynthetic proteins has been observed in *obgc-1* (*cpsar1*) RNAi mutants (Bang et al., 2012), indicating that they are potential cargos. Furthermore, recent studies using the *sco2*/*cyo1* mutant indicate that LHCB1 is a potential cargo (Tanz et al., 2012). However, SCO2/CYO1 interacts directly with LHCB1, but not with the components of the signal recognition particle (SRP) pathway that normally targets LHCB1 to the thylakoids. Moreover, the suggested pathway for LHCBs would mainly occur in the cotyledon stage and be less significant for mature plants (Tanz et al., 2012). This is consistent with the reported embryo lethality of *cpsar1* mutation (Bang et al., 2009; Chigri et al., 2009; Garcia et al., 2010) and the coloration of *obgc-1* (*cpsar1*) RNAi mutant leaves (pale green when young, but wild-type like in later developmental stages; Bang et al., 2012). If LHCBs are cargo proteins several more may be present, including (potentially) at least some of the LHCB member proteins that interact with CPRabA5e (Karim et al., 2014). Bioinformatics searches for homologs to thylakoid-located proteins with a COPII cargo selection motif have identified several candidates, including 21 putative transmembrane cargos and 12 putative soluble cargos (Khan et al., 2013). Interestingly, 45% of these potential cargo proteins are linked to photosynthesis, indicating that some nuclear-encoded proteins may be targeted to thylakoids via vesicles (in addition to the four other known targeting mechanisms; Spetea and Aronsson, 2012). Moreover, some of the putative transmembrane cargo proteins were LHCBs (Khan et al., 2013).

## IS THERE A COPII, COPI, OR CCV TRANSPORT SYSTEM INSIDE CHLOROPLAST?

If CPSAR1 is involved in vesicle transport in chloroplasts, in accordance with its localization and bioinformatics indications (Garcia et al., 2010; Brandizzi, 2011), homologs of components of other secretory pathways should be present in chloroplasts (unless the chloroplast system radically differs from other vesicle transport systems). Accordingly, some have been identified and the first outcomes were mainly COPII-related components, acting mainly in the vesicle formation stage (Andersson and Sandelius, 2004), but others (including GAPs, GEFs, tethering factors, and SNAREs) were identified in a more recent extended bioinformatics analysis (Khan et al., 2013). However, recent data (Lindquist et al., 2014) indicate that no system resembling cytosolic CCV or COPI systems is present in *Arabidopsis* or rice chloroplasts. Several chloroplastic proteins homologous to subunits of typical COPI and CCV systems were identified, but they have already been ascribed with other functions and unrelated to vesicle transport. About 30 chloroplastic proteins were identified in a motif-based homology search, including homologs for four B-COPI coat subunits and one F-COPI coat subunit. Even more CCV-related subunits were identified, including putative clathrin heavy and light chain components, one or more subunits of AP1-5 complexes, and some coat GTPases (Arf-related proteins). However, no homologs of several other essential proteins for a functional COPI or CCV vesicle transport systems were detected. Thus, the presence of a CCV- or COPI-like system in chloroplasts seems unlikely (unless, as mentioned, some components radically differ from their cytosolic counterparts). The occurrence of a putative CCV AP2 β2 subunit speculatively supports the idea of a unique system, since this subunit is present in yeast, but not in *Arabidopsis* cytosol. In addition, several of the putative COPI and CCV subunits identified in chloroplasts had greater resemblance to yeast than *Arabidopsis* counterparts. Thus, further analysis is required to determine definitively if vesicle transport in chloroplasts relies solely on COPII-related proteins, or a distinct system perhaps involving some proteins similar to cytosolic COPI/CCV proteins is present (Khan et al., 2013; Paul et al., 2014). Furthermore, although some proteins, possibly involved in a COPII-related vesicle transport system in chloroplasts, have been identified, their exact functions and localization remain to be clarified (Khan et al., 2013; Paul et al., 2014).

## CONCLUDING REMARKS

Proteins here described as being linked to chloroplast vesicle transport do share similar trends regarding vesicle transport and thylakoid formation and structure. In all chloroplast mutant phenotypes (except VIPP1 being responsible for vesicle initiation) observed vesicle accumulation inside chloroplasts implicate these proteins to have a role in vesicle transport. Moreover, they demonstrate similar role for thylakoid membrane formation and morphology. Thus, recent identification of putative chloroplast localized vesicle transport proteins and the empirical verification of the location and function of few of them provide strong indications that vesicle-based protein transport machinery is present in chloroplasts. However, numerous aspects have not yet been elucidated, notably the precise roles of the CPSAR1 and CPRabA5e GTPases, and other putative participants. Better knowledge of these systems and their actors would help efforts to understand evolutionary aspects of their acquisition by land plants and their roles in both cargo protein transport to the thylakoids and recycling back to or through the membranes. It could also help to elucidate important but poorly understood aspects of chloroplast biogenesis, e.g., thylakoid formation and maintenance, including building of the photosynthetic machinery.

## Conflict of Interest Statement

The authors declare that the research was conducted in the absence of any commercial or financial relationships that could be construed as a potential conflict of interest.
